# The potential role of human HIV-1 TAT-Interactive Protein 2 levels in the pathogenesis of contact dermatitis

**DOI:** 10.3906/sag-2106-81

**Published:** 2021-10-23

**Authors:** Mahmut Sami METİN, Handan BİLEN, Ömer Faruk ELMAS, Necmettin AKDENİZ

**Affiliations:** 1 Department of Dermatology, Faculty of Medicine, Kırşehir Ahi Evran University, Kırşehir, Turkey; 2 Department of Dermatology, Faculty of Medicine, Ataturk University, Erzurum, Turkey; 3 Department of Dermatology, Faculty of Medicine, Kırıkkale University, Kırıkkale, Turkey; 4 Department of Dermatology, Memorial Ataşehir Hospital, İstanbul, Turkey

**Keywords:** Contact dermatitis, CC3/TIP30, HTATIP2/TIP30

## Abstract

**Background/aim:**

Human HIV-1 TAT interactive protein 2 (HTATIP2/TIP30) is a gene that is extensively expressed in human tissues as well as in tumor tissues. This study aimed to explore the potential role of HTATIP2/TIP30 in contact dermatitis (CD), which is one of the most common inflammatory cutaneous conditions.

**Materials and methods:**

This cross-sectional study involved adult patients with acute contact dermatitis who were admitted to the outpatient dermatology clinic of a tertiary hospital and healthy adult volunteers without any cutaneous or systemic diseases. The blood concentration of HTATIP2/TIP30 was measured using ELISA kits.

**Results:**

The research sample consisted of 31 patients with CD (18 males, 13 females) and 20 healthy control subjects (14 males, 6 females). The mean ages of the patients with CD and healthy volunteers were 37 and 30 years, respectively (p > 0.05). The mean value of serum HTATIP2/TIP30 levels in patients with CD was 1.65 ng ml^−^^1^, which is 0.60 ng ml^−^^1^ in the control group (p = 0.02)

**Conclusion:**

In this study, serum levels of HTATIP2/TIP30 were statistically significantly higher in patients with CD when compared to healthy controls. This outcome may indicate possible role of HTATIP2/TIP30 in the pathogenesis of CD.

## 1. Introduction

Contact dermatitis (CD) is an inflammatory skin disease characterized by pruritus, erythema, vesicles, blisters, and scales. It can be in the form of acute, subacute, or chronic dermatitis. Approximately 80% of CD cases are irritant contact dermatitis (ICD) while 20% are allergic contact dermatitis (ACD). ICD is the results of localized, nonimmunological inflammatory reaction. ACD, unlike ICD, is a type 4 hypersensitivity reaction to a specific allergen that also results in a series of inflammatory reactions [1]. Although both forms of CD may have similar clinical presentations, they can be differentiated on pathophysiological grounds. Regardless of the pathophysiological pathway of two different forms, cutaneous inflammation is essential [2].

Human HIV-1 TAT interactive protein 2 (HTATIP2/TIP30), which is also called CC3/TIP30, is a pro-apoptotic protein expressed in different tumors. It has been described as related to the transcriptional activation area of HIV-1 Tat, and it is especially cofactor in Tat-activated transcription [3]. The role of TIP30 in cell death and tumor suppression is a well-known phenomenon. TIP30 and related factors have been shown to be involved in the control of gene expression associated with apoptotic and metastatic processes [4–9].

A wide variety of chronic inflammatory disorders or autoimmune conditions are associated with an increased risk of malignancy. The role of chronic inflammation in cancer development has been a subject of experimental and epidemiologic studies. It is hypothesized that chronic infection or persistent inflammation enforces de novo lymphogenesis and resulted in malignancy. In certain organs such as the lung and liver, chronic inflammation related to fibrosis can also be associated with malignant transformation [10–11]. To the best of our knowledge, no study has directly investigated the possible relationship between inflammatory conditions and HTATIP2/TIP30 expression. Almost all the relevant studies have focused on the role of HTATIP2/TIP30 in cancer pathogenesis. Uzkeser et al, however, investigated the serum HTATIP2/TIP30 levels in patients with rheumatoid arthritis, ankylosing spondylitis, and sarcoidosis, referring to the role of inflammation in the cancer pathogenesis. The authors found that serum HTATIP2/TIP30 levels in patients with ankylosing spondylitis were higher than in healthy controls. They concluded that this result may be associated with low apoptotic activity in patients with ankylosing spondylitis [12].

In this study, we aimed to investigate serum HTATIP2/TIP30 levels in patients with contact dermatitis, a prototypical cutaneous inflammatory condition.

## 2. Materials and methods

### 2.1. Design of the study, patient selection, and data collection

This cross-sectional study involved adult patients with acute contact dermatitis who were admitted to the outpatient dermatology clinic of a tertiary hospital between January 2014 and August 2015 and healthy adult volunteers without any cutaneous or systemic diseases. Patients with a cutaneous condition other than CD or a systemic disease were excluded. Subacute or chronic cases of CD were also rule out. The diagnosis of CD was made by the expert opinion of the author based on the clinical characteristics and detailed history. The control group consisted of age- and sex-matched healthy volunteers. All patients were newly diagnosed, and none of them received topical or oral corticosteroids before the diagnosis. After at least 8 h of fasting, 5 cc peripheral venous blood samples were collected into two blood collection tubes from all participants. Serum samples of CD and healthy controls were obtained after centrifugation and stored at −80 °C until the analysis date. The serum HTATIP2/TIP30 levels were tested using a HTATIP2/TIP30 enzyme-linked immunosorbent assay (ELISA) kit (Cusabio Biotech Co., Ltd. Catalog No: CSB-E14917H) in accordance with the manufacturer’s instructions.

### 2.2. Statistical analysis

Computerized statistics software (SPSS, Inc., Chicago, IL, USA) was used for statistical analysis. Descriptive data were given as mean ± standard deviation, number, or percentage. Unpaired Student’s t-test was used for comparisons of HTATIP2/TIP30 levels between the groups. The statistically significant level was set at p < 0.05. The actual power of the study was found to be 0.78. Power analysis was calculated using the G-Power (G*Power Version 3.1.9.6; Universitat Kiel, Germany) package program.

## 3. Results

**Figure 1:**
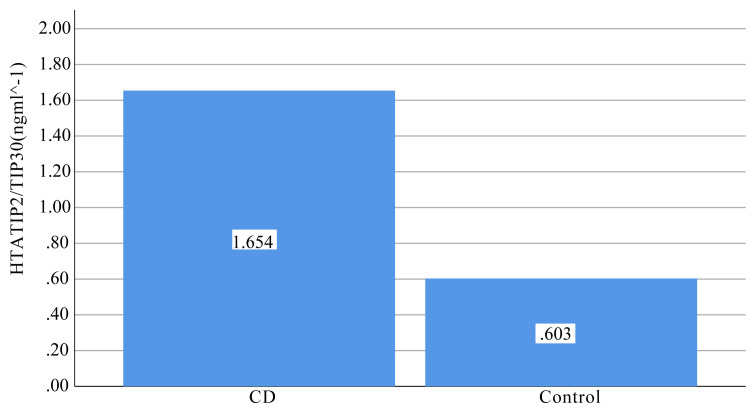
HTATIP2/TIP30 in blood of patients with CD and control group.

**Figure 2:**
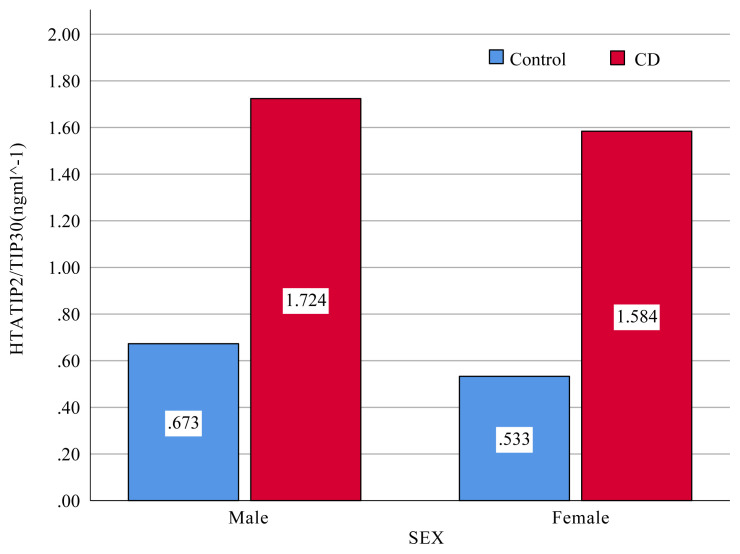
The paired t-test showed a significant difference between men and women in the control and patient groups in terms of average HTATIP2/TIP30 and evaluated using Student’s t test (t = 0.45, p < 0.05). Different letters indicate significant differences between means.

The research sample consisted of 31 patients with CD (18 males, 13 females) and 20 healthy control subjects (14 males, 6 females). The mean ages of the patients with CD and healthy volunteers were 37 (range 20–62 years) and 30 years (range 18–44 years), respectively. There were no differences between the groups in terms of age or sex (p> 0.05) (Table 1). The mean value of serum HTATIP2/TIP30 levels was significantly higher in patients group (1.654 ± 1.82 ng ml^−^^1^) compared to those of the controls (0.603 ±0.21 ng ml^−^^1^). (Table 2, Figure 1). In addition, HTATIP2/TIP30 levels were higher in men than women both in the control (p < 0.05) and patient groups (p < 0.05) (Figure 2).

## 4. Discussion

A limited number of studies investigated the role of HTATIP2/TIP 30 expression in various diseases [13]. In this study, we found that the levels of HTATIP2/TIP30 were significantly higher in patients with CD when compared to healthy subjects.

Contact dermatitis (CD) is a worldwide, frequent disease, which is responsible for about 10% of the visits to dermatology offices. CD expresses a group of cutaneous diseases caused by contact with allergens or irritants. It is characterized by different stages of an eczematous eruption. Irritant contact dermatitis (ICD) and allergic contact dermatitis (ACD) are considered as the two subgroups of the entity [14]. ACD is a type 4 or delayed hypersensitivity reaction. It occurs after exposure to an allergen, which activates T helper 1 cells in a previously sensitized individual. ICD, unlike ACD, is a nonimmunologic inflammatory reaction that can be seen in anyone who has been exposed to sufficient duration and number of irritant agents causing direct epidermal keratinocyte damage [15–17]. Although the causes of these two conditions may differ, both have quite similar clinical, histological, and molecular features.

Both innate and acquired immunity play roles in the immune response against cancer [18]. The pathology of inflammation comprises two main events that consisted of vascular and cellular events. During vascular events, interstitial edema occurs because of vascular leakage of exudate due to vasodilatation and increased vascular permeability. This is the sign of a “tumor” of inflammation. The most impressive functional alteration among cellular events is the migration of leukocytes to the area and secretion of their products into the medium [19]. HTATIP2/TIP30 is actually a normal cellular protein described as a tumor suppressor [20–21]. It is known that its secretion is augmented by heat shock proteins, stress, certain viruses (i.e. EBV, retrovirus), TGF-β, and aging [21]. Tumor suppressor activity of HTATIP2/TIP 30 is associated with suppressing a pro-inflammatory cytokine called osteopontin (OPN), which has an important role in cellular immunity and existed in T helper 17 (IL-17 producing T helper cells) along with blockage of nuclear pores and induction of apoptosis [22]. It has been shown that HTATIP2/TIP30 levels are decreased in variant-small cell lung carcinoma (V-SCLC), classic-SCLC, neuroblastoma (NB), colon cancer, melanoma, prostate cancer, and hepatocellular carcinoma (HCC) [23–24]. Liu et al. reported that the loss of function of TIP30 has been linked to metastasis in nonsmall cell lung cancer (NSCLC). In another study, researchers demonstrated that the absence of HTATIP2 expression in A549 human NSCLC cells resulted in accelerated tumor growth despite stalled tumor neovascularization and reduced tumor oxygenation, and lowered tumor sensitivity to sorafenib treatment.

Engkilde et al. reported that contact allergy was found to be associated with four different cancer subtypes. Most of the relationships were inverted, which may support the immune surveillance hypothesis. They found a meaningful and inverse connection between contact allergy and breast cancer and non-melanoma skin cancer, respectively, as well as a meaningful and positive connection between contact allergy and bladder cancer [25]. In patients with cancer, an immune response occurs against the tumor cell. However, this immune response is not sufficient for eradicating malignant cells or preventing metastasis. In these patients, foreign microorganisms such as bacteria or viruses are perceived as a signal of danger and generate a very strong immune response, whereas tumor cells are perceived as self-cells and a weaker immune response develops. In patients with cancer, a high amount of CD4+, CD25+ cells are detected in the circulation and around tumoral tissue. Not only tumor-related antigen-specific T cells but also nonspecific T cells, natural killer cells, and macrophages play role in the development of immune response against tumoral tissue [26]. Zhang et al. reported that interventional therapy for primary HCC has good short-term efficacy. This therapy reduces the levels of serum HTATIP2/TIP30, B7-H4, AFP, and inflammation-related indexes [27]. Certain chronic inflammatory disorders or autoimmune conditions are associated with an increased risk of malignancy. The role of chronic inflammation in cancer development has been a subject of experimental and epidemiologic studies [11,28]. Consequently, both innate and acquired immunity play role in the pathogenesis of the malignant diseases as well as that of CD. Although several studies have shown the role of HTATIP2/TIP30 in malignant diseases, there is no study assessing the potential role of HTATIP2/TIP30 in CD.

Our study has several limitations that need to be addressed. The first one is the relatively small number of participants. Second, the cross-sectional design of the study did not make it possible to remeasure HTATIP2/TIP30 levels after cutaneous inflammation had resolved. Third, it is hard to draw a robust conclusion on the relationship between HTATIP2/TIP30 and contact dermatitis, since tissue expression of HTATIP2/TIP30 and detailed immunological evaluations were not made.

In this study, we found significantly higher serum HTATIP2/TIP30 levels in patients with CD when compared to healthy controls. However, prospective studies with tissue expression analysis are warranted to demonstrate the potential role of HTATIP2/TIP30 in inflammatory events.

## Figures and Tables

**Table 1:** Some demographics properties in patient and control groups.

Parameters	Control	CD
Number	20	31
Age (range)	30.35 (18–44)	36.96 (20–62)
Sex	14 M/6 F	18 M/13F

**Table 2:** Serum HTATIP2/TIP30 levels in patient and control groups.

Groups	Number	Mean, ng ml^−1^	S.D.	p	max d	min d
Control	20(14M/6F)	0.603(0.673M/0.533F)	0.214	0.02*	1.09	0.36
Patient	31(18M/13F)	1.654(1.724M/1.584F)	1.828		9.32	0.37

* p < 0.05 significant level (unpaired t-test).

M: Male, F: Female, SD: Standard deviation.
